# Chronic Allergic Inflammation Causes Vascular Remodeling and Pulmonary Hypertension in Bmpr2 Hypomorph and Wild-Type Mice

**DOI:** 10.1371/journal.pone.0032468

**Published:** 2012-03-09

**Authors:** Elizabeth M. Mushaben, Gurjit Khurana Hershey, Michael W. Pauciulo, William C. Nichols, Timothy D. Le Cras

**Affiliations:** 1 Division of Pulmonary Biology, Cincinnati Children's Hospital Medical Center, Department of Pediatrics, University of Cincinnati, Cincinnati, Ohio, United States of America; 2 Division of Asthma Research, Cincinnati Children's Hospital Medical Center, Department of Pediatrics, University of Cincinnati, Cincinnati, Ohio, United States of America; 3 Division of Human Genetics, Cincinnati Children's Hospital Medical Center, Department of Pediatrics, University of Cincinnati, Cincinnati, Ohio, United States of America; Vanderbilt University Medical Center, United States of America

## Abstract

Loss-of-function mutations in the bone morphogenetic protein receptor type 2 (BMPR2) gene have been identified in patients with heritable pulmonary arterial hypertension (PAH); however, disease penetrance is low, suggesting additional factors play a role. Inflammation is associated with PAH and vascular remodeling, but whether allergic inflammation triggers vascular remodeling in individuals with BMPR2 mutations is unknown. Our goal was to determine if chronic allergic inflammation would induce more severe vascular remodeling and PAH in mice with reduced BMPR-II signaling. Groups of Bmpr2 hypomorph and wild-type (WT) Balb/c/Byj mice were exposed to house dust mite (HDM) allergen, intranasally for 7 or 20 weeks to generate a model of chronic inflammation. HDM exposure induced similar inflammatory cell counts in all groups compared to controls. Muscularization of pulmonary arterioles and arterial wall thickness were increased after 7 weeks HDM, more severe at 20 weeks, but similar in both groups. Right ventricular systolic pressure (RVSP) was measured by direct cardiac catheterization to assess PAH. RVSP was similarly increased in both HDM exposed groups after 20 weeks compared to controls, but not after 7 weeks. Airway hyperreactivity (AHR) to methacholine was also assessed and interestingly, at 20 weeks, was more severe in HDM exposed Bmpr2 hypomorph mice versus WT. We conclude that chronic allergic inflammation caused PAH and while the severity was mild and similar between WT and Bmpr2 hypomorph mice, AHR was enhanced with reduced BMPR-II signaling. These data suggest that vascular remodeling and PAH resulting from chronic allergic inflammation occurs independently of BMPR-II pathway alterations.

## Introduction

Pulmonary arterial hypertension (PAH) is a devastating disease characterized by vascular dysfunction and remodeling. Arterial remodeling causes increased pulmonary vascular resistance, resulting in sustained pulmonary artery pressures that lead to right ventricular hypertrophy and subsequently death due to right-sided heart failure [1]–[9]. Although PAH is a rare disease, once diagnosed, life expectancy is generally less than three years in adults [4], [6] and less than 1 year in children [10] without therapeutic interventions. Despite intense investigation into the pathogenesis of PAH, the etiology remains unclear and present therapies only slow disease progression [2], [4], [10].

Some insight into the pathogenesis of PAH has come from the discovery that heterozygous mutations in the bone morphogenetic protein receptor type 2 gene (BMPR2) are present in many patients with heritable pulmonary arterial hypertension (PAH) and some patients with idiopathic PAH [1], [11]–[15]. The BMPR2 gene encodes the bone morphogenetic protein receptor type II (BMPR-II), which is a member of the transforming growth factor-β superfamily and plays a critical role in embryogenesis, apoptosis, cell growth, and cell differentiation. Upon ligand binding, the type I BMP receptor is activated by BMPR-II, causing phosphorylation and activation of downstream signaling molecules, Smads 1, 5 and 8 [16]–[19]. While approximately 70% of patients with heritable PAH have mutations in the BMPR2 gene, the disease penetrance is low, with some reports showing that only ∼10–20% of family members with BMPR2 mutations actually develop symptomatic PAH [1], [5]–[7], [13], [14]. As a result, it has been suggested that other genetic and/or environmental factors may be necessary for clinical expression of PAH in individuals with BMPR2 mutations [14].

PAH is known to occur in diseases in which inflammation is a cardinal feature (i.e. autoimmune diseases and HIV infection), leading to the suggestion that inflammation may be an important mediator or trigger of pulmonary arterial remodeling [3], [20]. In addition, data from patients with PAH have demonstrated increases in inflammatory cells in the vicinity of remodeled vessels, as well as elevated levels of pro-inflammatory cytokines such as IL-1β and IL-6 [2], [20]–[22]. Experimental studies in mice and rats have shown that inflammatory models such as chronic hypoxia [23], [24], monocrotaline [21], [25], [26], and allergic asthma (ovalbumin, *Aspergillus fumigatus*, house dust mite) [3], [27], [28] all develop varying degrees of vascular remodeling. These data further support a role for inflammatory pathways in vascular remodeling and disease, however, the exact involvement of these pathways remains poorly defined.

Given the potential role of inflammation in vascular remodeling [3], [21], [23]–[27] and the development of PAH in patients with BMPR2 mutations [1], [11]–[15], we hypothesized that chronic inflammation would trigger more severe arterial remodeling and PAH in mice with reduced BMPR-II signaling. To address this question we utilized Bmpr2 hypomorph mice, as Bmpr2 null mice die during early gastrulation [29]. Bmpr2 hypomorph mice have a deletion in exon 2 (hereafter referred to as Bmpr2 ΔE2 mice) that encodes half of the extracellular ligand-binding domain of the receptor [30]. This deletion results in reduced BMPR-II signaling [30] similar to that seen in patients with BMPR2 mutations [4]. Bmpr2 ΔE2 mice that are homozygous for the hypomorphic allele die during mid-gestation with cardiovascular and skeletal defects: however, mice that are heterozygous for the hypomorphic allele develop into adulthood with no apparent abnormalities [30]. In this study, we exposed wild-type (WT) and Bmpr2 ΔE2 mice to the aeroallergen, house dust mite (HDM), for 7 or 20 weeks to induce chronic inflammation, as previous studies had reported vascular remodeling in WT mice following such a protocol [27]. Muscularization of pulmonary arterioles and right ventricular systolic pressures (RVSP) were measured to assess the severity of vascular remodeling and PAH after chronic inflammation in both groups. Additionally, airway hyperreactivity (AHR) was assessed as part of the allergic response and to assess airway function since a study had reported reduced BMPR-II expression in the airways of patients with asthma [31]. Vascular remodeling and mild PAH developed after 20 weeks of HDM in both Bmpr2 ΔE2 and WT mice; however, there was no difference between these groups. Interestingly, AHR was more severe after 20 weeks of HDM exposure in Bmpr2 ΔE2 mice compared to WT, demonstrating a phenotypic difference in these mice. These results suggest that vascular remodeling and PAH resulting from chronic allergic inflammation occurs independently of Bmpr2 mutations. The exaggerated AHR in Bmpr2 ΔE2 mice suggests a potential role for this pathway as a modifier of chronic allergic airway disease.

## Results

### Genotype Analysis of DNA from Bmpr2 ΔE2 Mice

**Figure 1:**
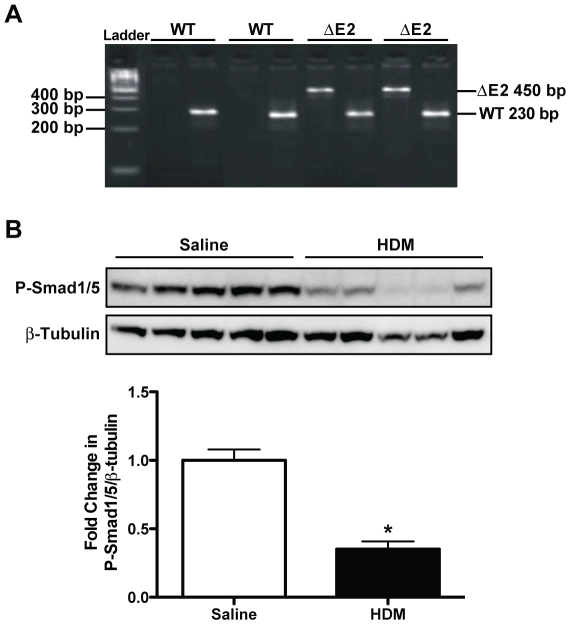
Genotype analysis of Bmpr2 hypomorph allele and levels of P-Smad1/5 in HDM exposed mice. **A**: Tail DNA from mice carrying the wild-type (WT) and/or the Bmpr2 hypomorph (ΔE2) alleles were genotyped by PCR using the primers Bmpr2 MT Neo R1 to detect the hypomorph allele (ΔE2 450 bp) and Bmpr2 WT R2 to detect the WT allele (WT 230 bp). PCR products were run on a 4% agarose gel containing ethidium bromide and photographed. **B**: Western blot analysis of P-Smad1/5, a downstream mediator of BMPR-II signaling, was decreased by approximately 50% after HDM exposure compared to saline controls (*n* = 5 mice/group). *P<0.05 vs saline.

To verify the genotype of the mice, PCR of tail DNA from WT and Bmpr2 ΔE2 mice was performed using primer probe sets specific for both the WT and hypomorph alleles. WT and Bmpr2 ΔE2 mice were distinguishable by the amplification of a 450-bp fragment for the hypomorph allele (Figure 1A).

#### Alterations in BMPR-II Signaling in Response to HDM

To determine if signaling downstream of BMPR-II was altered in response to HDM, western blot analysis for phosphorylated Smad1/5 (P-Smad1/5) was performed on lung homogenates of saline and HDM exposed WT mice. P-Smad1/5 levels were reduced by approximately 50% in mice exposed to HDM for 20 weeks compared to saline controls (Figure 1B).

### Inflammatory cell response and Immunoglobulin Levels after HDM Exposure

**Figure 2:**
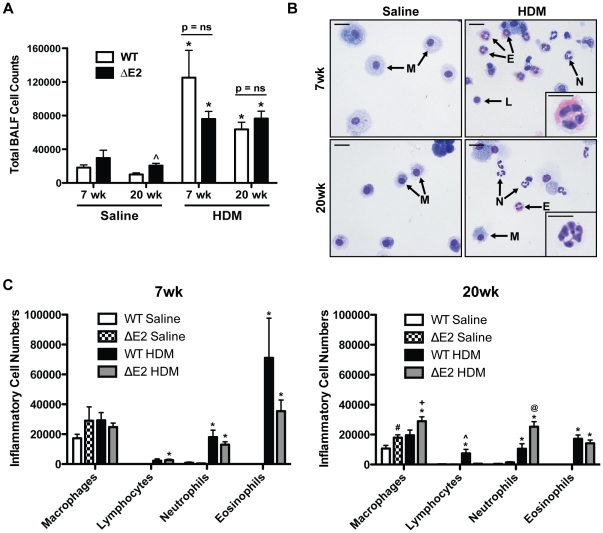
Inflammatory cell response in HDM exposed mice. **A**: Total inflammatory cell counts were increased in BALF in both WT (white bars) and Bmpr2 ΔE2 (ΔE2; black bars) mice after 7 weeks (wk) and 20 weeks of HDM (n = 4–13 mice/group in two independent experiments). *P<0.05 vs saline control, ∧P<0.05 vs saline WT 20 weeks. **B**: Representative images of cytospins from BALF demonstrating specific inflammatory cell types in the lung after 7 and 20 weeks of saline or HDM exposure. The inset in the top panel shows a high power image of an eosinophil and the bottom panel inset is a representative image of a neutrophil. M = Macrophage, L = Lymphocyte, N = Neutrophil, and E = Eosinophil. Scale bar larger panels = 20 µm. Insets scale bar = 10 µm. **C**: Changes in macrophage cell numbers were only observed after 20 weeks of HDM and only in BMPR2 ΔE2 mice. Neutrophils and eosinophil numbers were increased in HDM exposed groups at both time points, however, the number of eosinophils was lower after 20 weeks. *P<0.05 vs saline control, ∧P<0.05 vs saline ΔE2 lymphocyte 20 weeks, #P<0.05 vs saline WT macrophage 20 weeks, +P<0.05 vs HDM WT macrophages 20 weeks, @P<0.05 vs HDM WT neutrophil 20 weeks.

**Figure 3:**
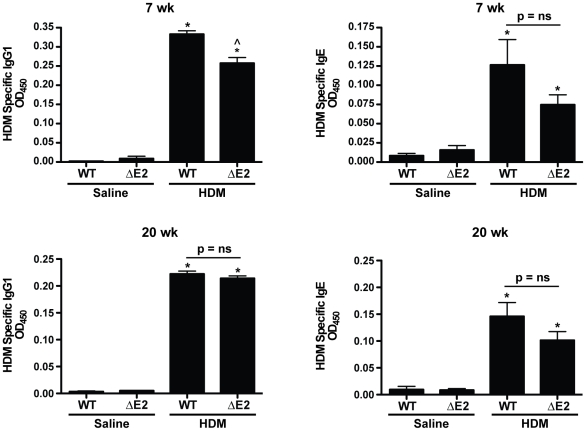
Allergic sensitization in HDM exposed mice. Levels of HDM specific IgG1 and IgE were increased in both WT and Bmpr2 ΔE2 mice after 7 weeks (top panels) and 20 weeks (bottom panels) of HDM exposure. At 7 weeks, HDM specific IgG1 levels in WT mice were slightly higher compared to Bmpr2 ΔE2 mice (n = 4–12 mice/group). *P<0.05 vs saline control, ∧P<0.05 vs HDM WT 7 weeks.

Total inflammatory cells were assessed in the BALF. Increases in total inflammatory cell counts were similar in HDM exposed WT and Bmpr2 ΔE2 mice, at both 7 and 20 weeks (Figure 2A). Specific inflammatory cell types were assessed by Diff-Quick staining of cytospins. Representative images are shown in Figure 2B. Total neutrophil and eosinophil numbers were increased after 7 weeks of HDM exposure compared to saline controls. After 20 weeks of HDM, neutrophil and eosinophil numbers were still increased above controls although total eosinophil numbers were lower compared to 7 weeks (Figure 2C). In addition, the percentage of neutrophils and eosinophils were increased in WT and Bmpr2 ΔE2 mice after 7 and 20 weeks of HDM exposure compared to saline controls (Table 1). Lymphocytes percentages were increased only in HDM treated Bmpr2 ΔE2 mice at 7 weeks, but similarly increased in both WT and Bmpr2 ΔE2 at 20 weeks. The percentage of eosinophils was lower and the percentage of neutrophils was increased after 20 weeks of HDM compared to 7 weeks. HDM specific IgG1 and IgE levels were measured after HDM exposure to assess sensitization to the allergen. HDM specific IgG1 and IgE levels were similarly increased in HDM exposed WT and Bmpr2 ΔE2 groups compared to saline controls after 7 (top panels) and 20 (bottom panels) weeks (Figure 3). HDM specific IgG1 was slightly higher (1.3 fold) in WT mice compared to Bmpr2 ΔE2 mice after 7 weeks of HDM exposure, but not after 20 weeks. Increases in HDM specific IgE were similar in WT and Bmpr2 ΔE2 mice at 7 and 20 weeks.

**Table 1:** Percentage of inflammatory cells in BALF of WT and Bmpr2 ΔE2 mice after saline or HDM exposure.

	Macrophages	Lymphocytes	Neutrophils	Eosinophils
**7 wks HDM**				
WT Saline	99.7±2.4%	0.0±0.0%	4.0±2.2%	0.3±0.2%
ΔE2 Saline	98.2±0.4%	0.1±0.1%	1.6±0.3%	0.1±0.1%
WT HDM	28.3±4.8%*	1.6±0.5%	17.3±3.5%*	52.9±6.5%*
ΔE2 HDM	35.5±5.3%*	3.4±0.7%*	17.2±1.5%*	44.0±4.8%*
**20 wks HDM**				
WT Saline	97.7±1.7%	0.8±0.4%	2.6±1.1%	0.1±0.1%
ΔE2 Saline	91.7±2.0%#	0.6±0.2%	7.6±1.9%@	0.1±0.0%
WT HDM	34.7±3.2%*	7.8±1.8%*	31.8±2.0%*	24.6±2.6%*
ΔE2 HDM	41.9±2.7%*	8.0±1.5%*	32.4±2.1%*	17.8±1.7%* ∧

Increases in the percentage of neutrophils and eosinophils were observed in both HDM exposed wild-type (WT) and Bmpr2 ΔE2 (ΔE2) mice after 7 wk of HDM compared to saline controls. Lymphocytes were also increased after 7 wk of HDM exposure in Bmpr2 ΔE2 mice. Lymphocytes, neutrophils, and eosinophils were all increased after 20 wk of HDM exposure compared to saline controls. Data are presented as mean ± SEM. N = 4–12 mice per group in two independent experiments.

* P<0.05 vs saline controls,

∧ P<0.05 vs WT HDM 20 wk eosinophils,

# P<0.05 vs WT saline 20 wk macrophages,

@ P<0.05 vs WT saline 20 wk neutrophils.

### Pulmonary Vascular Remodeling

**Figure 4:**
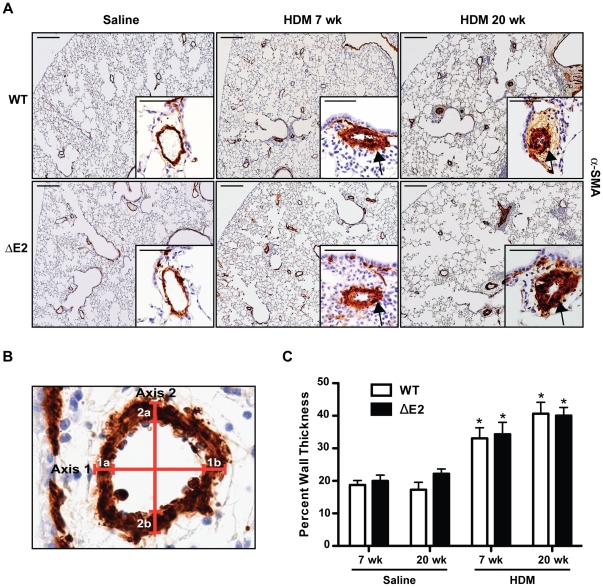
Vascular remodeling and arterial wall thickness following chronic HDM exposure. **A**: α-Smooth muscle actin (α-SMA) staining was performed on lung sections from WT (top) and Bmpr2 ΔE2 (ΔE2; bottom) mice exposed to saline or HDM for 7 or 20 weeks (wk). Thickening of the arterial wall (medial layer) in HDM exposed mice is seen (arrows) at both 7 and 20 weeks. Scale bar larger panels = 200 µm. Insets scale bar = 50 µm. **B**: Image of α-SMA immunostaining of a pulmonary artery demonstrating the method used to measure arterial wall thickness. For each vessel, external diameter and two wall thicknesses were measured along two different axes. Percent wall thickness was calculated as [(1a+1b)/external diameter]×100. This measurement was performed along each axis. The two axes measurements were averaged together to determine the final wall thickness. **C**: Percent wall thickness of pulmonary arteries (20–150 µm) was increased 1.5 fold in HDM exposed mice after 7 weeks (wk) and 2 fold in mice exposed to HDM for 20 weeks. Differences between WT (white bars) and Bmpr2 ΔE2 (ΔE2) (black bars) mice exposed to saline or HDM were not significant (n = 5–13 mice/group). *P<0.05 vs saline control.

**Figure 5:**
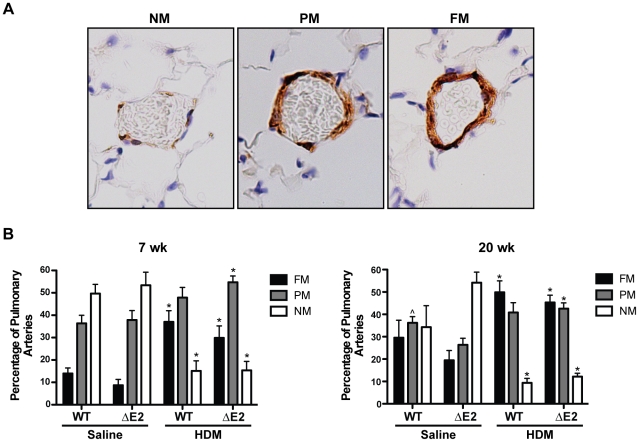
Muscularization of small pulmonary arteries. **A**: Representative images of α-SMA immunostaining of non-muscular (NM), partially muscularized (PM) [53], and fully muscularized (FM) small arterioles used to determine percent muscularization of vessels after HDM exposure (40×). **B**: WT and Bmpr2 ΔE2 (ΔE2) mice demonstrated increases in the number of arterioles that were fully muscularized (FM) and fewer arterioles that were non-muscular (NM) compared to saline controls after 7 weeks (wk) HDM (left). The percentage of FM arterioles was further enhanced after 20 weeks of HDM and the proportion of NM arterioles was further reduced. No differences were detected between groups of WT and Bmpr2 ΔE2 mice exposed to saline or HDM (n = 5–13 mice/group). *P<0.05 vs saline control, ∧P<0.05 vs ΔE2 saline PM.

No differences in thickening of the arterial wall of the small pulmonary arteries were detected between saline exposed WT and Bmpr2 ΔE2 mice (Figure 4A). Animals exposed to HDM for 7 or 20 weeks demonstrated increased thickening of the medial layer in small pulmonary arteries (20–150 µm) in both WT and Bmpr2 ΔE2 mice compared to saline controls (Figure 4A). Vessel wall thickness was measured by morphometric analysis as demonstrated in Figure 4B. Morphometric analysis revealed significant increases in wall thickness after HDM exposure; however, they were similar between WT and Bmpr2 ΔE2 mice at 7 and 20 weeks (Figure 4C). Mice exposed to HDM had a higher percentage of fully muscularized vessels compared to saline treated controls at both 7 and 20 weeks (Figure 5B). The percentage of fully muscularized vessels was further increased after 20 weeks of HDM exposure compared to 7 weeks. This pattern of muscularization was similar between HDM exposed WT and Bmpr2 ΔE2 mice at both time points. In addition to the increases in percentages of muscularized vessels, the total number of muscularized vessels (fully+partially together) was increased in HDM exposed mice compared to saline controls (data not shown).

### Pulmonary Arterial Hypertension

**Figure 6:**
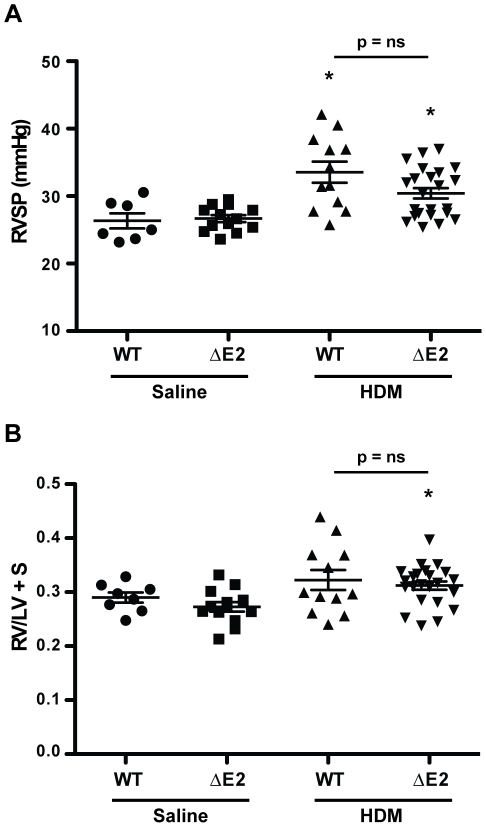
Pulmonary arterial hypertension following chronic HDM exposure. **A**: Right ventricular systolic pressures (RVSP) was increased in both WT and Bmpr2 ΔE2 (ΔE2) mice after HDM exposure for 20 weeks compared to saline controls. There was no difference in RVSP between WT and Bmpr2 ΔE2 mice exposed to HDM (n = 4–11 mice/group in two independent experiments). *P<0.05 vs saline control. **B**: The ratio of RV weight to LV+S weight was only increased in Bmpr2 ΔE2 mice compared to saline controls (n = 4–11 mice/group in two independent experiments). *P<0.05 vs saline control.

Right heart catheterization was performed to assess the development of PAH. In animals exposed to 7 weeks of HDM, no increase in right ventricular systolic pressure (RVSP) in either group was detected compared to saline controls (data not shown). Following 20 weeks of HDM exposure, however, RVSP was increased in WT (33.54±1.57 versus 26.34±1.11) and in Bmpr2 ΔE2 (30.41±0.76 versus 26.67±0.51) mice compared to saline controls indicating the development of mild PAH in these animals (Figure 6A). RVSP was not different between HDM exposed Bmpr2 ΔE2 mice and WT mice (30.41±0.76 versus 33.54±1.57). Although RVSP was increased in both HDM exposed WT and Bmpr2 ΔE2 mice after 20 weeks, the RV to LV+S weight ratio was only increased in Bmpr2 ΔE2 mice compared to saline controls (0.31±0.01 versus 0.27±0.01) (Figure 6B). However, there was no difference between the RV to LV+S weight ratio in HDM exposed WT and Bmpr2 ΔE2 mice (0.32±0.02 versus 0.31±0.01).

### Airway Hyperreactivity

**Figure 7:**
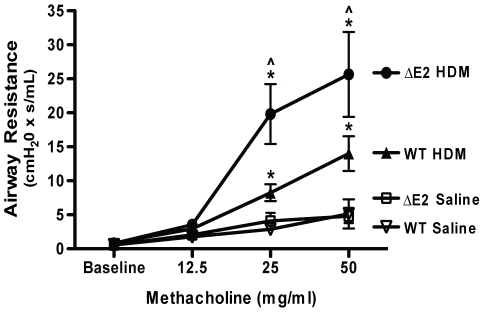
Airway hyperreactivity following chronic HDM exposure. Bmpr2 ΔE2 mice developed more severe airway respiratory resistance to methacholine after 20 weeks of HDM compared to WT mice (n = 4–12 mice/group). Both HDM groups demonstrated increased airway resistance with methacholine exposure compared to saline controls. *P<0.05 vs saline controls, ∧P<0.05 vs WT HDM.

After RVSP measurements were obtained, airway hyperreactivity (AHR) to increasing doses of methacholine was assessed since it had been previously reported that Bmpr2 expression was decreased in asthmatic patients [31]. After 7 weeks of HDM exposure, AHR was similarly increased in both WT and Bmpr2 ΔE2 mice compared to saline controls (data not shown). After 20 weeks of HDM exposure, AHR was increased in both WT and Bmpr2 ΔE2 compared to saline controls. Interestingly though, after 20 weeks, AHR was more severe in the HDM exposed Bmpr2 ΔE2 mice at both 25 mg/ml (2.4 fold) and 50 mg/ml (1.8 fold) methacholine compared to the HDM exposed WT mice (Figure 7).

## Discussion

The present study demonstrates that while mice exposed to the aeroallergen HDM for 7 weeks develop increases in wall thickness and muscularization of pulmonary arterioles, this was not associated with significant increases in RVSP. However, when HDM exposure was extended out to 20 weeks, arterial remodeling was more severe and RVSP was significantly increased, although mild. Despite these increases in RVSP and arterial remodeling, Bmpr2 ΔE2 mice showed similar responses to WT mice, suggesting that reductions in BMPR-II signaling do not predispose for more severe PAH with chronic allergen-induced inflammation. Although vascular changes were similar between Bmpr2 ΔE2 and WT mice, AHR in Bmpr2 ΔE2 mice was more severe after 20 weeks HDM which suggests a potential role for this pathway as a modifier of reactive airway disease.

Previous studies have examined the role of inflammation and chronic hypoxia in the development of PAH in Bmpr2 heterozygous mice (Bmpr2^+/−^). Zhang et. al. treated Bmpr2^+/−^ mice with adenovirus-delivered 5-lipoxygenase (5-LO), which is known to facilitate inflammation and is increased in patients and animal models of PAH [32], [33]. These studies demonstrated increased RVSP and vascular remodeling in Bmpr2^+/−^ mice compared to WT; however, the degree of muscularization in the 5-LO treated mice was mild [34]. A subsequent study demonstrated that Bmpr2^+/−^ mice treated with combined 5-LO and monocrotaline developed sustained increases in RVSP, thickening of small pulmonary arteries, and perivascular inflammation of the remodeled vessels that was more severe in Bmpr2^+/−^ mice compared to WT [26]. Another study utilizing Bmpr2^+/−^ mice, found that a combination of hypoxia and chronic serotonin infusion increased RVSP, right ventricular hypertrophy, and pulmonary remodeling [35]. In the present study, we utilized HDM, as many people develop allergic responses this common aeroallergen [27], [36], [37]. In addition, Erjefalt et. al. had previously demonstrated that chronic HDM exposure could cause pulmonary vascular modeling in mice after 20 weeks of HDM; however, no direct measures of PAH were reported [27]. Unlike reports mentioned earlier, in the present study, PAH severity was not enhanced in BMPR2 mutant mice compared to WT mice. This discrepancy may be due to the different types of inflammation induced in the previous studies since they primarily elicit Th1 responses and little to no eosinophils [20], [24], [26]. In contrast to previous models, HDM exposure induces the release of Th2 cytokines (interleukin 4 (IL-4), IL-5, and IL-13) and an influx of eosinophils during acute exposures and neutrophils during chronic exposures [27], [38]–[42]. A role for Th2 cytokines in the etiology of vascular remodeling was demonstrated by Grunig et. al. as pulmonary arterial remodeling in OVA and *Aspergillus fumigatus* exposed mice was reduced in IL-4 deficient mice. Comparing the findings of our study to others, suggests that the type of stimuli may be important in determining whether PAH is more severe or not in BMPR2 mutant mice. In addition, in some of these studies, more than one insult was required for animals with reduced BMPR2 to show any PAH phenotype [26], [35], suggesting that the development of the disease may be multi-factorial. This is consistent with additional genetic and/or environmental factors being necessary to trigger PAH in patients with BMPR2 mutations since the penetrance of the disease is low.

In the present study, we assessed vascular remodeling and RVSP after 7 and 20 weeks of HDM exposure to determine if the changes in arterial remodeling were associated with PAH. In addition, we examined the question of whether a Bmpr2 signaling deficiency in HDM exposed mice would cause more severe pulmonary arterial remodeling changes, since mutations in this gene are found in 70% of patients with heritable PAH. Although increases in arterial muscularization in HDM exposed mice were observed after 7 weeks, RVSP was not increased. However, after 20 weeks of HDM exposure, RVSP was similarly increased in both WT and Bmpr2 ΔE2 mice in conjunction with more severe arterial remodeling. Our data agrees with that of two previous studies which showed that persistent allergic inflammation could induce pulmonary vascular remodeling [3], [27], however; in addition, we were able to detect PAH, although mild. The development of PAH in our model might be partly due to a higher percentage of fully muscularized pulmonary arteries at 20 weeks compared to 7 weeks; however, factors such as vasoconstriction could also be playing a role in the pathophysiology.

Although an increase in the number of inflammatory cytokines, macrophages, T cells, B cells, and chemokines have been observed in patients with PAH, the exact role of these molecules in the disease process is unclear [3], [22], [43], [44]. As previously mentioned, Grunig et al. [3], exposed mice to ovalbumin and *Aspergillus fumigatus* for extended periods of time, and found that both of these allergens caused pulmonary arterial remodeling; however, increased muscularization of the pulmonary arteries did not correlate with RVSP measurements. A more recent study by Morrell et al. reported severe pulmonary arterial remodeling in the presence of Th1 and Th2 inflammatory responses in a mouse model of schistosomiasis, which is thought to be a common cause of pulmonary hypertension [45]. PAH was not observed in these mice, even though extensive pulmonary arterial remodeling was detected. In contrast to the studies above, we were able to detect increases in RVSP after inducing chronic inflammation in our model of vascular remodeling. Recent reports indicate that both mouse strain and sex can affect the inflammatory responses [46], [47]. It is possible that the strain of mice used (Balb/c/Byj) in this study may have influenced the response to chronic inflammation differently compared to the other studies since mice of a different background were used (C57BL/6) [3], [26], [35]. Although differences in sex have previously been shown to affect the inflammatory response, we were unable to detect any differences in inflammation or RVSP between males and females in our model; however, there was a trend towards higher RVSP in females.

In addition to vascular remodeling and PAH, we also assessed AHR in our model since a study reported decreased BMPR2 in the airways of asthmatic patients, providing evidence for a potential role for the BMPR2 pathway in allergic asthma [31]. Interestingly, in our study, more severe AHR was observed in Bmpr2 ΔE2 mice after 20 weeks of HDM exposure compared to WT mice. These findings offer further support that this pathway may be a modifier of the asthmatic response, although additional studies are needed to address this more extensively. Prior to this study, there had been little evidence suggesting any common features between PAH and asthma. One case report showed both increased pulmonary pressures and reactive airway disease in two patients with congenital heart disease [48]. After long-term treatment of these patients for the asthma symptoms, pulmonary pressures decreased; however, whether these two diseases are linked is unclear. One explanation for the lack of information regarding any connection between these two diseases may be due to the fact that the PAH patient population is small.

In summary, we demonstrated that chronic HDM exposure causes arterial remodeling and PAH in mice, although we were unable to detect any differences in the response between WT and Bmpr2 ΔE2 mice. This suggests that chronic HDM exposure causes vascular remodeling and PAH through mechanisms that may be independent of the BMPR-II signaling pathway. To our knowledge, this is the first study to establish that chronic allergen exposure causes PAH, albeit mild. Additionally, we observed more severe AHR in Bmpr2 ΔE2 mice compared to WT after chronic HDM exposure, suggesting a potential role for Bmpr2 in allergic airway disease.

## Methods

### Ethics statement, animal treatments, and RVSP measurements

Animal protocols and procedures were approved by the Animal Care and Use Committee at the Cincinnati Children's Hospital Research Foundation (Cincinnati, OH) (Protocol Number: 1D02011) and all procedures were performed under anesthesia to minimize suffering. Bmpr2 ΔE2 heterozygous mice were generated by Dr. Karen Lyon's lab (UCLA, Los Angeles) [30]. At Cincinnati Children's Hospital, mice were bred onto a mixed Balb/c/Byj background, since this strain responds well to allergens and is frequently used in allergic inflammatory models. All WT mice used were littermate controls from these breedings.

At 6–10 weeks of age, WT and Bmpr2 ΔE2 mice were exposed intranasally to either 20 µl saline (0.9% NaCl) (vehicle control group) or 25 µg of the allergen, house dust mite (HDM) (Greer Laboratories, Lenoir, NC), dissolved in 20 µl of saline, 3 times per week and for either 7 or 20 weeks. Isoflurane was used to anesthetize the mice during repeated intranasal HDM exposures. At the end of the treatment periods, mice underwent right heart catheterization, as previously described [34]. Briefly, mice were anesthetized with 2.5% isoflurane and remained sedated with ∼2.0–2.25% isoflurane via a nose cone during catheterization. After dissection to expose the right jugular vein, an SPR-671 pressure transducer catheter (Mikro-Tip 1.4F, Millar Instruments, Houston, Texas) was inserted into the jugular vein via a small incision, passed through the right atrium and into the right ventricle where right ventricular systolic pressures (RVSP) were measured. For each mouse, an average of 30 individual RVSP measurements during a 3–5 minute time period were taken and analyzed using the HSE Haemodyn W software (version 1.1.1.131, Harvard Apparatus).

### Genotype Analysis

Genotypes were determined by PCR (Figure 1) with the primer Bmpr2 WT R2 to detect the wild-type allele (230-bp) and Bmpr2 MT Neo R1 used to detect the neo cassette, which was inserted in place of exon 2 to generate a hypomorph allele (450-bp). Another primer, Bmpr2 F2, was used as a forward primer for both sets of reactions. Amplification was performed for 34 cycles with 1 minute at 95°C, 1 minute at 56°C, and 1 minute at 72°C. The sequences for the primers were: Primer Bmpr2 MT Neo R1, 5′ CCTTCTATCGCCTTCTTGAC 3′; Primer Bmpr2 WT R2 5′ TTCCCTGATAACAGCCTTCC 3′; Primer Bmpr2 F2 5′ AAGTACACGGTCGCTGTCTTC 3′.

### Western Blot Analysis

Western blot analysis was performed on lung homogenates using the following antibodies to assess the levels of phosphorylated Smad1/5 (P-Smad1/5, 1∶1,000; Cell Signaling) and β-tubulin (1∶1,000; Cell Signaling). A goat anti-rabbit secondary antibody (1∶10,000; Calbiochem) was used along with an ECL plus chemiluminescence detection system (GE Heathcare). An LAS4000 imaging system and Multi Gauge 3 software (Fujifilm, Tokyo, Japan) were used to image and quantitate the chemiluminescent signal for each Western blot. Protein loading and transfer efficiency were controlled for each sample by normalizing to β-tubulin.

### Airway Hyperreactivity

After RVSP measurements, mice were anesthetized with an intraperitoneal injection of Ketamine/Xylaxine/Acepromazine (4∶1∶1) solution and changes in airway resistance to methacholine were assessed as previously described [49]. Briefly, after a tracheostomy was performed, the mouse was connected to a flexiVent system (SCIREQ, Montreal, QC, Canada). Airway respiratory resistance was measured after nebulization of phosphate buffered saline (1× PBS) (baseline) and then increasing doses of methacholine (12.5, 25, and 50 mg/mL; acetyl-β-methylcholine chloride, Sigma, St. Louis, MO).

### Bronchoalveolar lavage

After AHR measurements were performed, bronchoalveolar lavage fluid (BALF) was collected from mice to assess the inflammatory response. Lungs were lavaged three times via a tracheostomy tube with a total volume of 1 ml of 1× PBS as previously described [49]. The BALF was centrifuged (2000 RPM) and the supernatant was removed and used to measure HDM specific IgE and IgG1 levels. Red blood cell lysis buffer (R7757 Sigma, St. Louis, MO) was added to the cell pellet to lyse any red blood cells. Cells were centrifuged again, supernatant removed, and cells resuspended in PBS. Total inflammatory cells were counted using a hemocytometer. Cytospins of the remaining cells were collected on slides for differential cell counts. Inflammatory cell types were determined by Diff-Quick staining (Shandon Lipshaw, Pittsburg, PA) (Figure 2B). Three hundred cells were counted per slide and the percentages of macrophages, lymphocytes, neutrophils, and eosinophils were determined.

### ELISA

HDM specific IgG1 and IgE levels were measured in BALF by ELISA to assess allergic sensitization to HDM as previously described [50]. Briefly, plates were coated with 0.01% HDM in PBS overnight. The following day, plates were blocked with 1% BSA in PBS for 1 hour then coated with samples. Biotin-anti-mouse IgE or IgG1 (Pharmingen, 2.0 µg/ml) was used for capture. Streptavidin-HRP (1∶100, R & D, Minneapolis, MN) was added to detect antibodies and the reaction developed by TMB substrate (1∶1) (BD Biosciences, San Jose, CA).

### Immunohistochemistry and arterial remodeling

Lungs were inflation fixed by tracheal installation of 4% paraformaldehyde at constant pressure (25 cmH_2_O), transferred to 70% ethanol after 24 hrs, cut into three sections, and embedded in paraffin as previously described [51]. Immunostaining for α-smooth muscle actin (α-SMA) was performed on 5 µm paraffin-embedded sections by incubating slides with an α-SMA monoclonal antibody (1∶10,000 dilution, Clone 1A4; Sigma) overnight at 4°C, followed by a goat anti-mouse IgG2a secondary antibody (1∶200, Southern Biotech, Birmingham, AL). A Zeiss Axioplan 2 microscope (Carl Zeiss Microimaging, Thornwood, NY) was used to obtain digital images of the immunostaining. All genotypes and identification numbers of the animals were blinded to the observer, and the images were randomized. Vessel wall thickness was measured on pulmonary arteries with an external diameter ranging from 20–150 µm that were associated with terminal bronchioles and fully muscularized. The total number of vessels measured ranged from 20–56 per group. Metamorph imaging software (v6.2; Universal Imaging/Molecular Devices, Downington, PA) was used to measure the medial wall thickness and external diameter of each vessel. For each vessel, wall thickness and the external diameter were measured twice, along two different axes (Figure 4B). Percent wall thickness was calculated as [(wall 1a thickness+wall 1b thickness)/external diameter 1]×100. This calculation was performed for each axis. Each axis measurement was averaged together to determine the final wall thickness. Four to six vessels were measured for each animal. In addition to wall thickness measurements, percent muscularization of the pulmonary arterioles was also assessed. Small pulmonary arterioles in the terminal bronchial and the alveolar regions were identified and scored for muscularization as previously described [52]. Briefly, vessels were scored as nonmuscular (NM) (<50% muscle around vessel), partially muscularized (PM) [53] (>50% but <100% muscle surrounding the vessel), or fully muscular (FM) (muscle surrounds 100% of vessel) (Figure 5A). Thirty small pulmonary arteries were scored per animal and expressed as a percentage of total vessels counted.

### Statistical Analysis

Statistical analysis was performed with Prism 4 software (GraphPad Software, San Diego, CA). Unpaired *t*-tests, one-way, and two-way ANOVA with Tukey's post-hoc test were used to make comparisons. P<0.05 was considered statistically significant.
